# Two doses of fosaprepitant included prophylactic treatment for the three-day cisplatin-based chemotherapy induced nausea and vomiting

**DOI:** 10.1007/s00432-024-05766-7

**Published:** 2024-06-05

**Authors:** Yanying Li, Yuming Wan, Xiaoyun Yang, Ping Chen, Yan Gui, Lang He, Yingwei Xie, Jing Tian, Ping Duan, Guangguo Liu, Yu Sun, Jiang Zhu

**Affiliations:** 1 Division of Thoracic Tumor Multimodality Treatment and Department of Medical Oncology, Cancer Center, West China Hospital, Sichuan University, No. 37, Guoxue Lane, Chengdu, 610041 China; 2 Cancer Center, West China Hospital, Sichuan University, Chengdu, China; 3 Department of Medical Oncology, Shangjin Nanfu Hospital, West China Hospital, Chengdu, China; 4 Panzhihua University Affiliated Hospital, Panzhihua, China; 5 Lung Cancer Ward of Chengdu 7th People’s Hospital, Chengdu, China; 6 Department of Oncology, Affiliated Hospital of North Sichuan Medical College, Nanchong, China; 7 Department of Oncology, The Fifth People’s Hospital of Chengdu, Chengdu, China; 8 Department of Oncology, The People’s Hospital of Dachuan Section, Dazhou, China; 9 Department of Oncology, The People’s Hospital of Leshan, Leshan, China; 10 Department of Oncology, Chengdu First People’s Hospital, Chengdu, China; 11 No.404 Hospital, Mian Yang, China; 12 Radiotherapy Physics & Technology Center, Cancer Center, West China Hospital, Sichuan University, No. 37, Guoxue Lane, Chengdu, China

**Keywords:** Fosaprepitant, Two doses, Three-day cisplatin, CINV

## Abstract

**Purpose:**

Neurokinin 1 receptor antagonists included prophylactic treatment was recommended for patients who receive one-day cisplatin chemotherapy. It is unclear whether the prolonged administration of fosaprepitant is effective for three-day cisplatin-based chemotherapy induced nausea and vomiting (CINV). We aim to explore the prophylactic antiemetic efficacy and safety of two doses of fosaprepitant included regimen in the patients receiving multiple-day cisplatin chemotherapy.

**Methods:**

This randomized, parallel-group, open-labelled study was conducted in nine hospitals between February 2021 and February 2023. Patients diagnosed as lung cancer and chemotherapy naive were screened. Eligible participants were scheduled to be treated with highly emetogenic chemotherapy regimen which including three days of cisplatin. Then they were randomly divided into the experimental group (two doses of fosaprepitant, Group 2DF) and the control group (one dose of fosaprepitant, Group C). The primary endpoints included the safety and the average none CINV days (NCDs). This study was registered on the website of chictr.org.cn, number ChiCTR2100042665.

**Results:**

Overall, 204 participants were randomly assigned, and 198 patients were analyzed. No statistical difference in adverse events was found between the two groups. All treatment-related adverse effects for fosaprepitant observed were of grade 1–2. The average NCDs of Group 2DF was significantly more than Group C (18.21 ± 3.40 days vs 16.14 ± 5.20 days, *P* = 0.001). Furthermore, the better life function score was achieved in Group 2DF according to FLIE questionnaire.

**Conclusion:**

The administration of two-dose fosaprepitant was safe and more effective than one dose in protecting patients from CINV induced by three-day cisplatin included chemotherapy.

**Supplementary Information:**

The online version contains supplementary material available at 10.1007/s00432-024-05766-7.

## Introduction

Platinum-based chemotherapy is an essential treatment strategy for lung cancer (Yuwen et al. 2019; Rosell and Karachaliou 2015). However, side effects induced by cytotoxic therapy are significant and varied (Rosell and Karachaliou 2015). Chemotherapy-induced nausea and vomiting (CINV) is regarded as a common and unpleasant adverse event patients encounter (Wu et al. 2018; Sommariva et al. 2016; Ranganath et al. 2015; Basch et al. 2017). Moreover, it was reported that the incidence of CINV is nearly 90% if patients receive a highly emetogenic chemotherapy (HEC) regimen including cisplatin (Wu et al. 2018; Jordan et al. 2017). A phase III trial demonstrated the efficacy of the single dose of 150 mg fosaprepitant was non-inferior to three-day aprepitant in patients who received HEC (Grunberg et al. 2011). This conclusion was also confirmed by another non-inferiority phase III study in China (Yang et al. 2017). Therefore, current guidelines recommended combining fosaprepitant, dexamethasone, and 5-HT_3_ receptor antagonist as the typical prophylactic antiemesis regimen for CINV induced by HEC (Jordan et al. 2017; Razvi et al. 2019).

Multi-day cisplatin included chemotherapy is widely used for the treatment of varied malignancies such as germ-cell tumors, sarcomas, lymphomas, et al. (Abdel-Malek et al. 2017; Kamiya et al. 2021; Kondagunta and Motzer 2006). A reality also exists that clinicians in China usually divide the total cisplatin dose to small doses for consecutively multiple days in order to improve the tolerance and reduce the renal toxicity (Furugaki et al. 2009). A prior real-world data from this study team showed a poor situation of CINV prevention in the patients receiving HEC (most were 3-day cisplatin included regimen) or moderately emetogenic chemotherapy (MEC) in Sichuan China (Sun et al. 2021), another observational study found that up to 68.8% of patients still exhibited a durable CINV after receiving three-day cisplatin-based chemotherapy (Zhu et al. 2018). Prophylaxis of CINV induced by multiple-day cisplatin must be improved.

For single-day chemotherapy, most guidelines have recommended detailed daily antiemetic regimens (Razvi et al. 2019), but for multi-day chemotherapy, there is no clear recommendation on how to administer prophylactic antiemetic drugs every day. A phase 3 study found that the prolonged administration of aprepitant (125 mg on days 1–3, 80 mg on days 4–7) had better control of CINV in acute and delayed phase for patients receiving five-day cisplatin therapy (Albany et al. 2012). It was also confirmed in another study that the usage of aprepitant for more than three days was effective and safe in multiple-day cisplatin regimen (Olver et al. 2013). We previously conducted a randomized controlled study and found that the 6-day administration of aprepitant was safe and more effective than the standard regimen of 3-day aprepitant in preventing CINV caused by 3-day cisplatin regimens (Li et al. 2022).

However, due to the difficulty in taking oral medication, some patients can only choose intravenous antiemetic agents. We conducted this clinical trial to evaluate the safety and effects of two doses of fosaprepitant in preventing CINV during the whole cycle of three-day cisplatin-based chemotherapy.

## Methods

### Patients

This was a multicenter, parallel-grouped, randomized trial which was conducted in nine cancer departments, Sichuan, China from February 2021 to February 2023. The study was approved by the ethic committee of West China hospital, Sichuan University and each participating institution. All participants entered the trial were fully informed and signed the written informed consent. The registration number was ChiCTR2100042665 on the website of chictr.org.cn.

Patients were included if they aged from 18 to 75 years with a pathological diagnosis of lung cancer, who were prior chemotherapy-naïve with ECOG (Eastern Cooperative Oncology Collaboration) physical status scored 0–2. They were scheduled to be treated with HEC regimens containing 3-day cisplatin. Additionally, the eligible patients should have a life expectancy more than 3 months and adequate cognitive ability to read, understand, and complete independently the study questionnaires and diaries.

Patients were excluded if they have gastrointestinal or brain metastases, or other chronic gastrointestinal disease which leading to recurrent nausea and vomiting. Patients were also excluded if the laboratory parameter of important organ function cannot meet the requirements of chemotherapy, if they were scheduled to receive radiotherapy, immunotherapy or targeted treatment within 21 days after the beginning of the chemotherapy, if patients with uncontrolled cancer pain which need to adjust the dose of opioids, or the digestive tract reaction of opioids has not been completely relieved. Besides, the presence of nausea and vomiting caused by whatever reason occurred within three days were also excluded.

### Randomization and masking

Enrolled patients were randomly assigned 1:1 (no masking of participants) either to the experimental group (Group 2DF: fosaprepitant 150 mg once on days 1 and 3) or control group (Group C: fosaprepitant 150 mg on day 1) according to a random assignment schedule (Proc plan process) created by the statistical software SAS (Version 9.4). The randomization was balanced by using random blocks (4 participants per block) with stratification according to gender and chemotherapy regimen. The statistician who was not involved in the trial used the Interactive Web Response System (IWRS) to allocate participants to each group.

### Procedures

The patients in Group 2DF received the prophylactic antiemetic regimen as follows: fosaprepitant 150 mg iv one hour before cisplatin on day 1 and day 3, oral palonosetron 0.5 mg on day 1, and oral dexamethasone 6 mg on day 1, 3.75 mg on day 2–4. Patients in Group C were administered 150 mg fosaprepitant iv on day 1, oral palonosetron 0.5 mg on day 1, and oral dexamethasone 6 mg on day 1, 3.75 mg on day 2–4. The prophylactic antiemetic therapy was not specifically required in the subsequent cycles of chemotherapy.

Enrolled patients were asked to complete a diary based on MAT, which was a scale to access the occurrence of CINV. The diary was used to record the frequency of vomit, the degree of nausea (evaluated by VAS scale), rescue therapy, adverse effects, and the change in appetite from day 1 to 21 after chemotherapy. Rescue therapy was stated as any medication which was prescribed for treating the established nausea or emesis (Wu et al. 2018). The period from the day of chemotherapy begins until the time of appetite returns to normal and stable was recorded as the time of appetite recovery. If the patient was without any appetite loss, it was recorded as 0 day. However, those who could not reach the normal appetite were identified as 21 days. Meanwhile, patients needed to complete the Functional Living Index-Emesis (FLIE) questionnaire on the 8th day and 22th day. We used MAT on day 4, 8, and 22 to explore the CINV condition in different periods. Assessment of safety was performed after collection of adverse events which was rating by Common Terminology Criteria for Adverse Events (CTCAE 5.0).

In the current study, acute CINV was defined as the occurrence of nausea and/or vomit during day 1–3, while the delayed CINV phase was defined as day 4–7. The period from day 8 to day 21 was the time beyond the risk phase.

### Outcomes

The primary endpoints included the safety and none CINV days (NCDs). The NCD was identified as the very day that the participant was protected from any vomit, nausea, or rescue therapy in a whole day time (Li et al. 2022). The sum total of NCD in the period from day1-21 was the NCDs, data was extracted from the patient’s diary and the medical record.

The secondary endpoints included the following: (1) total control of CINV in the acute phase, the delayed phase and beyond the risk phase; (2) time to attain total control of CINV; (3) patients’ life function status; (4) the time of appetite recovery; (5) no impact on daily life (NIDL) (defined as total score > 108 by the FLIE questionnaire). Moreover, total control of CINV was defined as no vomiting, no nausea (the maximum VAS score less than 5 mm) and no prescription of the rescue therapy (Li et al. 2022). The period from the day of chemotherapy started to the last day of CINV occurred was regarded as the time to attain total control (Li et al. 2022).

### Statistical Analysis

According to our previous clinical study of prolonged aprepitant administration, the NCDs of the control group with 3-day aprepitant was 13.58 days (Li et al. 2022). In this study, the experimental group was expected to increase NCDs by 2 days compared with the control group. Assuming that the NCDs of the experimental group was 15.58 days, the two-sided test level α was 0.05, and the power level was 0.80, a total of 208 patients were required to be enrolled. Considering the loss to follow-up rate of 1%, we calculated 212 participants would need to be enrolled.

The statistical analyses in this study were performed by SPSS 19.0 Version. Three indexes including the NCDs, the appetite recovery time, as well as the FLIE score were evaluated by using T test. The time to total control of CINV was analyzed by Kaplan–Meier method and log-rank test. Additionally, the chi-square test was used to examine the adverse effects, total control, no nausea, no vomiting, and NIDL (Li et al. 2022). The statistical analyses were all performed on a double-sided test, and the statistical test level is α = 0.05 (Li et al. 2022). *P* < 0.05 was regarded as statistically significant.

## Results

### Patients’ characteristics

**Fig. 1:**
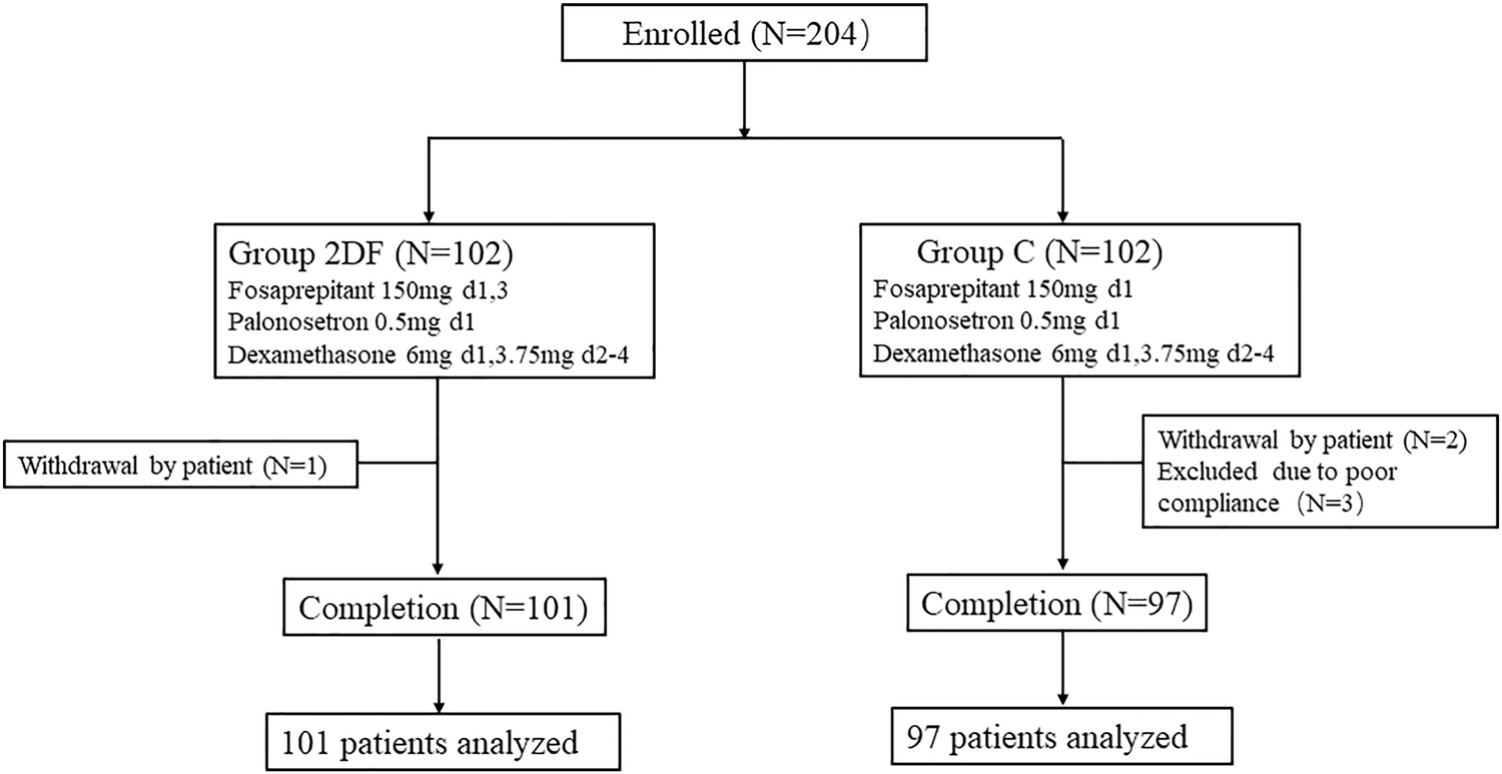
Flow chart of the study

When we had enrolled 204 patients, we completed the interim analysis with a positive result, so we did not enroll further patients. 102 patients were randomized into experimental group, whereas 102 were grouped into control group. 3 cases withdrew informed consent, 3 cases were excluded, and a total of 198 cases completed the study process and were included in statistical analysis (101 patients in experimental group, 97 in control group) (Fig. 1).

The result showed no significant difference between the two groups in patients’ baseline characteristics, including the known risk factors for CINV such as female, history of alcohol use and chemotherapy regimens (Table 1).

**Table 1:** Patients’ baseline characteristics

	Group 2DF	Group C	*P*-value
	(n = 101)	(n = 97)	
Gender			0.072
Male	62	70	
Female	39	27	
Age(years)			0.153
Median (range)	60 (30–75)	59 (37–70)	
ECOG PS			0.323
0 score	49	53	
1	46	42	
2	6	2	
Pathology			0.374
NSCLC	89	83	
SCLC	12	14	
Chemotherapy regimen			0.699
Pemetrexed and Cisplatin	56	50	
Paclitaxel and Cisplatin	33	32	
Etoposide and Cisplatin	12	14	
Gemcitabine and Cisplatin	0	1	
The history of alcohol	21	19	0.487

*ECOG PS* Eastern Cooperative Oncology Group performance status, *NSCLC* non-small cell lung cancer, *SCLS* small cell lung cancer

### Safety

198 patients were ultimately included in the safety set, of whom 143 occurred adverse events (experimental group: 71/101; control group: 72/97). No study-related death occurred and no fosaprepitant related serious adverse events (SAEs) occurred. Besides, all treatment-related adverse effects (TRAEs) for fosaprepitant observed were of grade 1–2. The TRAEs for fosaprepitant (hiccups, erythema at the injection site, constipation, diarrhea, headache) were similar in the two groups (Table 2).

**Table 2:** Summary of TRAE

	Group 2 DF (N = 101)	Group C (N = 97)	*P* value
All adverse events	71 (70.3%)	72 (74.2%)	0.724
Serious TRAEs	0	0	1.000
Commonly reported TRAEs (≥ 1% of subjects)			
Hiccups	5 (5.0%)	4 (4.1%)	1.000
Erythema at the injection site	0	1 (1.0%)	0.490
Constipation	6 (5.9%)	5 (5.2%)	1.000
Diarrhea	3 (3.0%)	4 (4.1%)	0.717
Headache	2 (2.0%)	0	0.498
Commonly reported AEs (≥ 10% of subjects)			
Myelosuppression	25 (24.8%)	24 (24.7%)	0.820
Anemia	17 (16.8%)	18 (18.5%)	0.683
Fatigue	29 (28.7%)	34(35.1%)	0.363

*TRAEs* treatment-related adverse events, *AE* adverse event

### None chemotherapy induced nausea and vomiting days (NCDs)

**Fig. 2:**
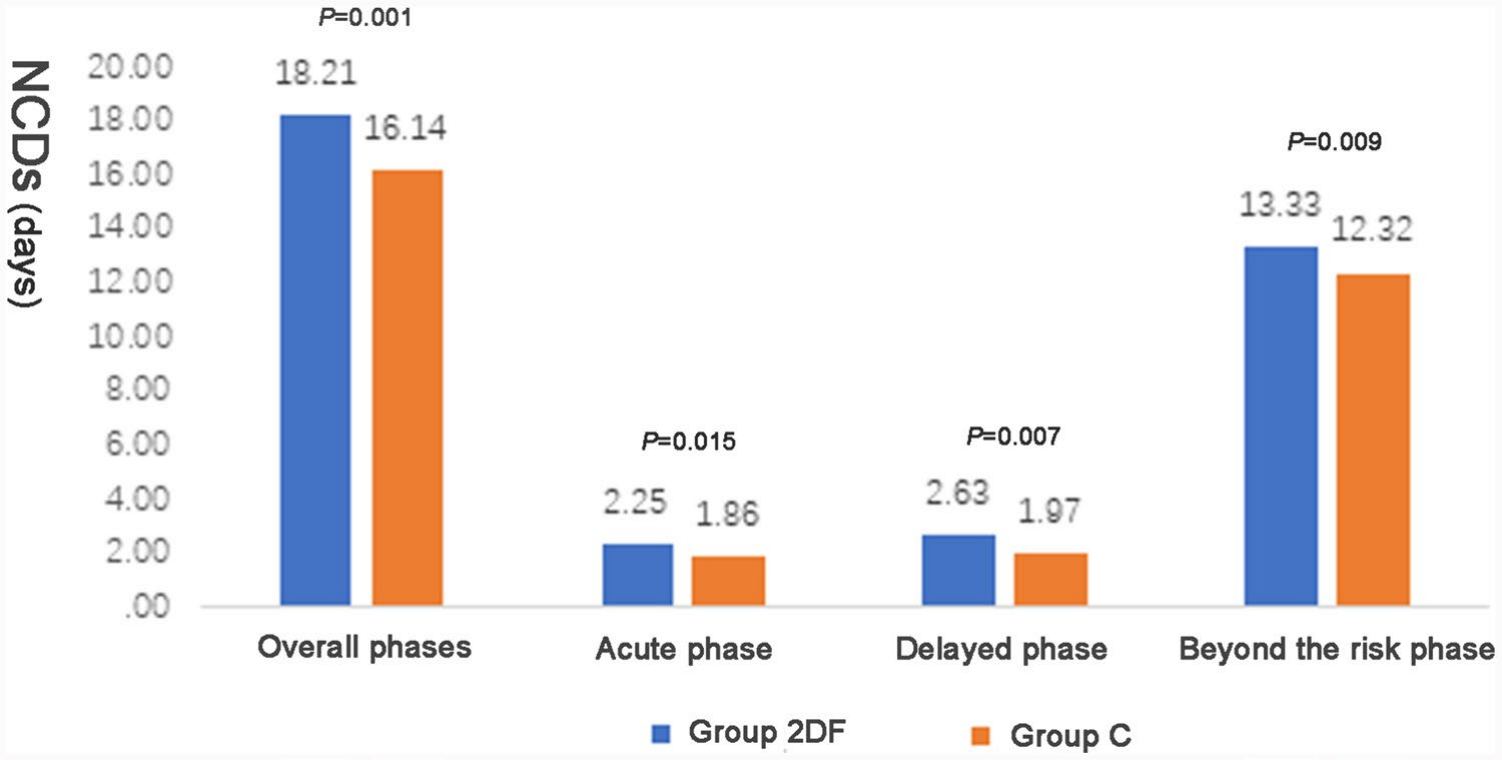
None CINV days (NCDs) in different phases

The average NCDs of Group 2DF were significantly more than those of Group C (18.21 ± 3.40 days vs. 16.14 ± 5.20 days, *P* = 0.001) in a whole chemotherapy cycle. The average NCDs of Group 2DF was significantly longer than that of Group C in the acute CINV phase, the delayed CINV phase and the period beyond the risk phase (Fig. 2).

### Total control of CINV

The ratio of CINV total control in Group 2DF was significantly higher than that in Group C during the acute and delayed phase (59.4% vs. 46.4% *P* = 0.045, 54.5% vs. 38.1% *P* = 0.015, respectively), and a better trend was observed in Group 2DF beyond the risk phase (80.2% vs. 69.1% *P* = 0.051). In the acute period, while no statistical difference was observed in the no vomiting between the two groups, no nausea in Group 2DF was better. Group 2DF exhibited higher rates of no nausea and no vomiting compared to the control group in the delayed phase. No significant difference was observed in any of these percentages between the two arms beyond the risk phase (Table 3). Throughout the whole chemotherapy cycle, 8 patients (7.90%) in Group 2DF received rescue antiemetic therapy, while 14 patients (14.40%) did in Group C (*P* = 0.109).

**Table 3:** CINV total control

	Group 2DF (n = 101)	Group C (n = 97)	*P*-value
Total control			
Acute	60 9(59.4%)	45 (46.4%)	0.045
Delayed	55 (54.5%)	37(38.1%)	0.015
Beyond the risk period	81 (80.2%)	67 (69.1%)	0.051
No nausea			
Acute	60 (59.4%)	45 (46.4%)	0.045
Delayed	57 (56.4%)	38 (39.2%)	0.011
Beyond the risk period	82 (81.2%)	69(71.1%)	0.067
No vomiting			
Acute	92 (91.1%)	82 (84.5%)	0.116
Delayed	88(87.1%)	70 (72.2%)	0.007
Beyond the risk period	93(92.1%)	87 (89.7%)	0.368

The median time to total control of CINV in Group 2DF were 4 days (95% CI 0.21–7.79 days), which was significantly shorter than that in Group C (7 days 95% CI 6.06–7.94 days,* P* = 0.019).

### Patients’ life function

**Fig. 3:**
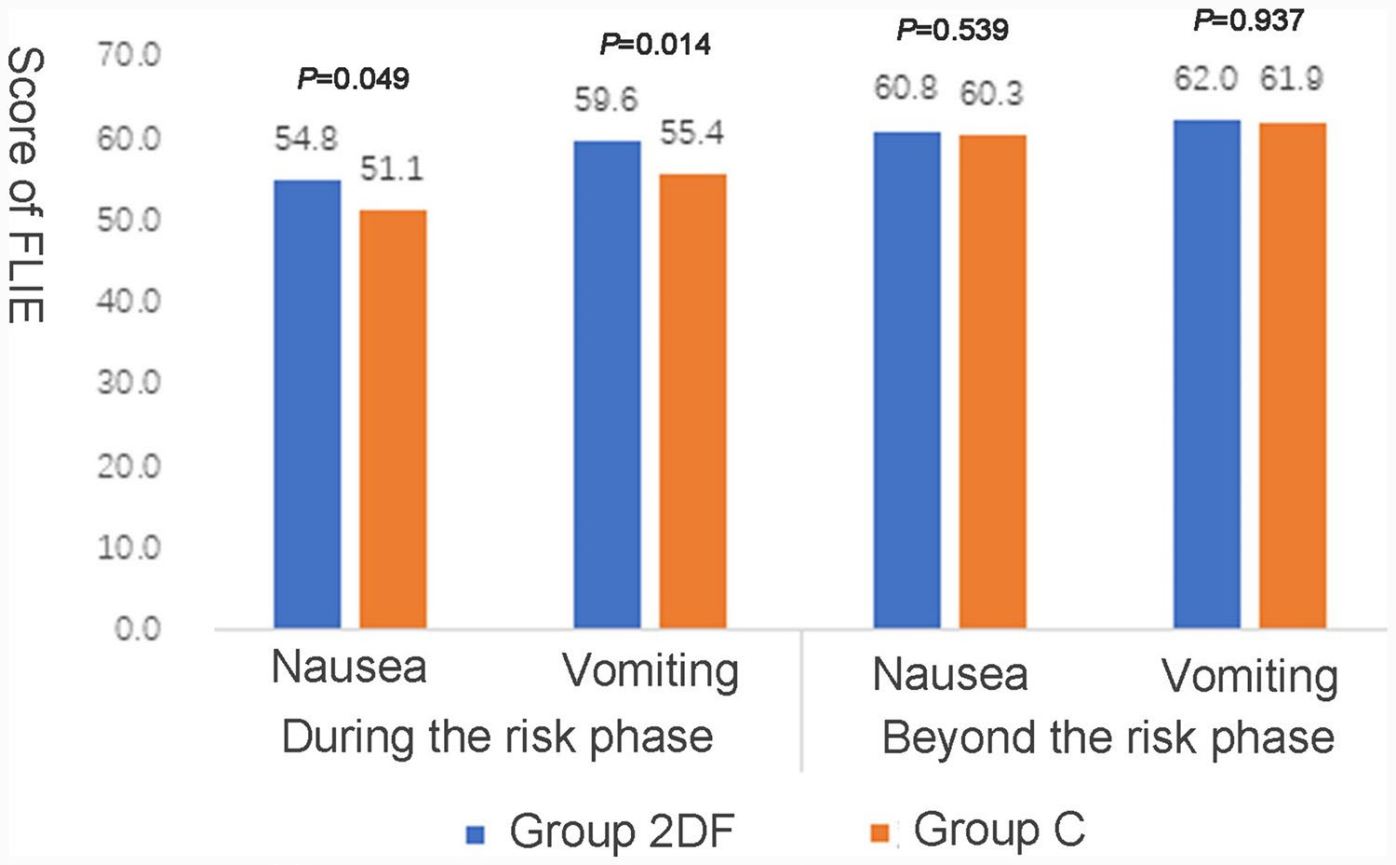
Score of FLIE in different phases

A better functional daily life was observed in the experimental group from the data in the FLIE questionnaire. Further analysis found the patients in group 2DF were less influenced by nausea or vomiting compared with the control group within the risk period (the average FLIE score: 54.8 *vs.* 51.1, *P* = 0.049; 59.6 vs. 55.4, *P* = 0.014, respectively). However, the functional life influence from nausea or vomiting had no significant difference between the two groups beyond the risk phase (the average FLIE score: 60.8 vs. 60.3, *P* = 0.539; 62.0 vs. 61.9, *P* = 0.937, respectively) (Fig. 3).

Moreover, the NIDL due to nausea, vomiting, or CINV in Group 2DF were all higher than that in the control group during the risk period respectively (66.3% *vs.* 53.6%, *p* = 0.046, 89.1% *vs.* 71.1%, *p* < 0.001, 77.2% vs*.* 62.9%, *p* = 0.020). However, the benefit did not persist beyond the risk phase (Table 4).

**Table 4:** Proportion of no impact on daily life accounting for the Functional Living Index–Emesis results in patients experiencing nausea, vomiting, and overall combined domains

	Group 2DF (n = 101)	Group C (n = 97)	*P*-value
CINV risk period (day 1–7)			
NIDL due to nausea	67 (66.3%)	52 (53.6%)	0.046
NIDL due to Vomiting	90 (89.1%)	69 (71.1%)	0.001
NIDL due to CINV	78 (77.2%)	61 (62.9%)	0.020
Beyond the risk period (day 8–21)			
NIDL due to nausea	90 (89.1%)	85 (87.6%)	0.459
NIDL due to Vomiting	96(95%)	92 (94.8%)	0.601
NIDL due to CINV	96 (95%)	88 (90.7%)	0.182

NIDL: No impact of daily life; CINV: Chemotherapy-induced nausea and vomiting

### Time to appetite recovery

**Fig. 4:**
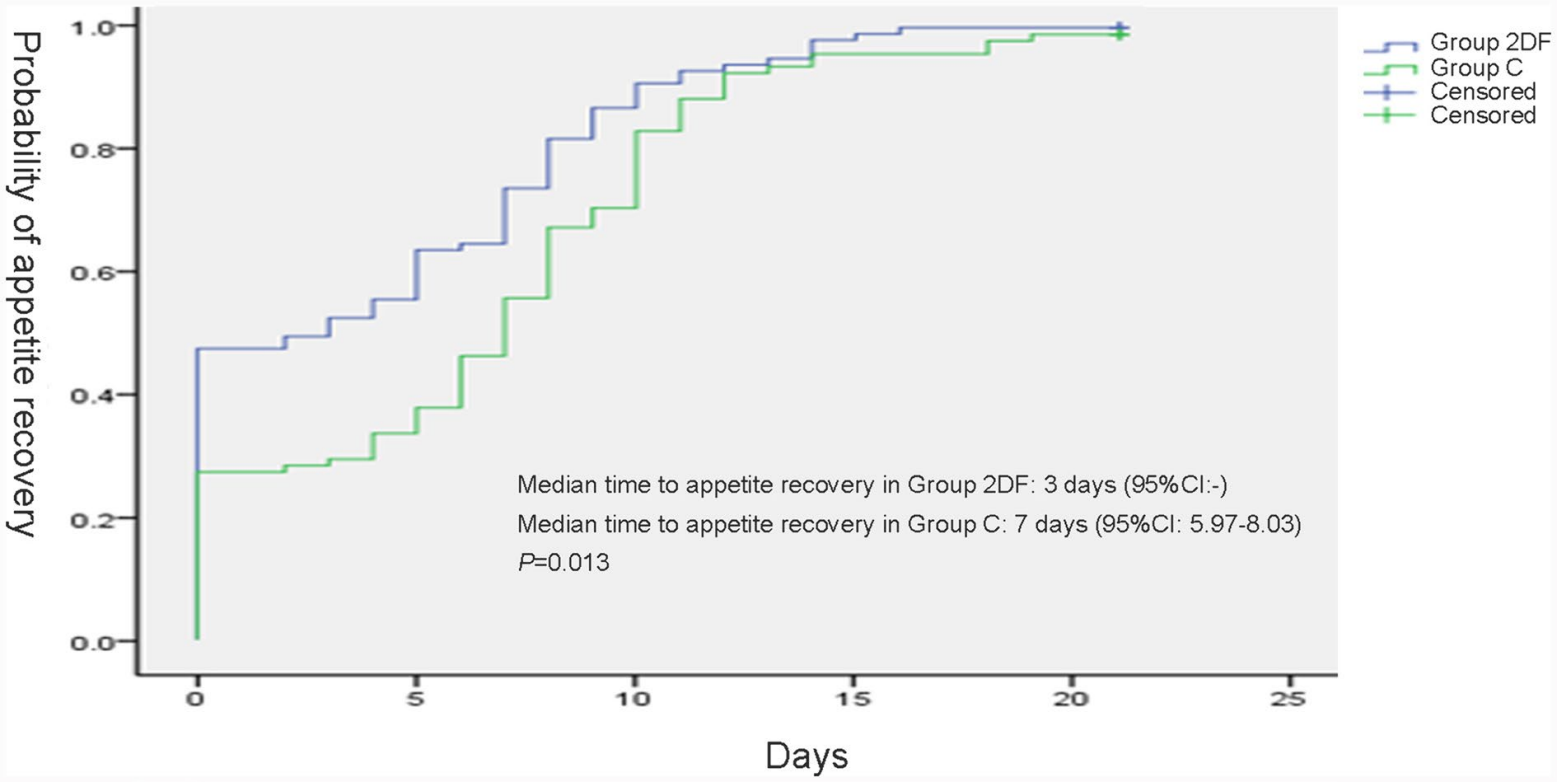
Time to appetite recovery

The median time to appetite recovery in Group 2DF was 3 days (95% CI: -), which was significantly shorter than that in Group C (7 days 95% CI 5.97–8.03 days, *P* = 0.013) (Fig. 4).

## Discussion

Multiday cisplatin included HEC has been widely used in various malignances, especially germ cell tumors, lymphomas, sarcomas, et al. Due to the higher risk of renal toxicity and the requirement of hydration process for single day cisplatin, clinically, the administration of cisplatin is usually split to three days (Zhang et al. 2020). Additionally, for patients with poor tolerance or expected high risk of chemotherapy, multiple cisplatin is frequently considered (Funai et al. 2010).

Due to the prolonged administration of cisplatin, patients will suffer simultaneously the acute and delayed CINV, and the duration of nausea and vomiting may last longer. Thus, the character of multiday-cisplatin induced CINV should significantly differ from that of the single-day cisplatin chemotherapy regimen. There are no clear recommendations for prophylactic antiemetic regimens of multi-day chemotherapy in various guidelines. Therefore, it is clinically significant to explore optimal prevention and control strategies for CINV induced by multiday chemotherapy. To date, the acute (the first day of chemotherapy) and the delayed (day 2–5) CINV phases have been used as critical research phase in most clinical studies (Wu et al. 2018; Schwartzberg et al. 2018; Hashimoto et al. 2020; Zhang et al. 2018). CINV occurred beyond the risk phase was investigated in our previous studies (Sun et al. 2021; Li et al. 2022, 2018; Wei et al. 2023), and the incidence of CINV occurred during 120-168 h was about 26% according to the recent Japanese data (Hata et al. 2022). Researchers tried the novel definition of CINV phase for multi-day cisplatin chemotherapy: the current study team defined the acute phase as day1–3, the delayed phase as day4 -day7, and the period beyond the risk phase as from day 8 to 21 in our prior studies about 3-day cisplatin (Li et al. 2022; Wei et al. 2023). Another Chinese trial focused on 3-day cisplatin regimen defined the acute phase as day1-day3, the delayed phase as day4-day8 (Zhao et al. 2023). In our study, the acute phase of CINV was defined as day1-3, the delayed phase as day 4–7, while the CIVN beyond the risk period was identified as day 8–21 of the chemotherapy cycle.

This study was designed to repeat fosaprepitant administration on day 3 for a possible better protection against CINV, the safety of double doses of fosaprepitant brings one of the most concerns. The results showed the repeat administration of fosaprepitant had comparable and acceptable safety profiles compared with the control group. Treatment-related death was not occurred in the experimental group, and all AEs were graded 1–2. These data demonstrated no significant difference from control group. Moreover, no specific reaction was observed in experimental group. In contrast, repeat administration of fosaprepitant showed satisfying safety. The above data of safety is similar to the prior studies (Gao et al. 2023). Based on the available data, it appears to be safe to increase the frequency or the duration of NK1RAS, and no significant risk of aggravating the chemotherapy side effects had been observed.

Patient’s diaries played an important role in this study, and it made it possible for us to access to detailed, unabridged and subjective information about patients’ CINV in the whole chemotherapy cycle, even the patients were at home. So, we can confirm that persistent CINV occurred during multiple days beyond risk period, not just limited in the first seven days that called acute or delayed CINV. This reality indicates that we should focus on not only “CINV risk period”, but CINV in the whole chemotherapy cycle also worthy of attention in the further studies. The study team regarded NCDs as an essential index to reflect the veritable situation of CINV within the whole chemotherapy cycle which could calculate accurately from the participants’ diary (Li et al. 2022). As observed, Group 2DF achieved an average NCDs as 18.21 ± 3.40 days, which meant that the patients receiving two doses of fosaprepitant would been protected from influence of CINV for more than 18 days in the whole 21-day chemotherapy cycle. Moreover, we found the good protection was occurred not only in the acute and delayed phase, but also beyond risk period. The protective phenomena in acute and delay phase are propose to be related to the repeat administration of fosaprepitant on day 3. Interestingly, neither group received prophylactic antiemetic strategy for CINV beyond risk phase, but NCDs remained higher in the experimental arm. This finding proposes that repeat fosaprepitant administration can extend prophylactic antiemetic effects for days, and make a better effect throughout the whole chemotherapy cycle.

We also analyzed the daily rate of acute nausea and vomiting control during day 1 to day 3. A significant higher rate of nausea and vomiting control was observed in Group 2DF on day 3 compared to the control group (67.3% vs 47.4%, P = 0.004). While no statistically significant was found on day 1 and day 2 between the two group. It was indicated that the better control on day 3 was related to the repeat dose of fosaprepitant.

The total control rate of CINV in Group 2DF was significantly better than that in Group C in the acute and delayed phase. The result showed the time to total control of CINV and recovery of appetite in the experimental group was shorter, indicating that repeat fosaprepitant administration can help the patients break away from CINV quickly and overcome the CINV impact sooner.

Additionally, the FILE scale was used to evaluate the impact of CINV on patients’ daily life within the chemotherapy cycle. It was observed that the participants in the experimental arm achieved better quality of life during risk period just as the prior study of prolonged use of aprepitant (Li et al. 2022). NIDL due to nausea, vomiting and CINV of Group 2DF were better than Group C during the risk period. But there was no difference between the two groups beyond the risk period. The above results indicate that more than two doses or on-demand administration of fosaprepitant might do better job, further study is considered. The experimental group had better NCDs beyond the risk period, but this did not lead to better life function. The proportion of patients who achieved complete control between the two groups had the greatest difference in the first week of chemotherapy, and this difference gradually narrowed, to the last week of chemotherapy cycle, less than 10% of patients in both groups did not achieve complete control and the difference was similar. This study reviewed life function on days 8–21 but filled out the FLIE scale only on day 22, as forgetting over time may not adequately reflect the overall situation of the two weeks. The absence of significant differences in NIDL beyond risk period between the two groups may be related to the similarity of complete control rates between the two groups in the last week.

Finally, are two doses of fosaprepitant the best regimen for patients receiving 3-day cisplatin chemotherapy? Is it necessary to administer more doses of fosaprepitant? In this study, some patients had experienced long-term persistent CINV, could persistent CINV be controlled by continuing to increase the number of doses of fosaprepitant? Hence, further data is needed.

## Conclusions

 Two doses of fosaprepitant included prophylactic antiemetic strategy showed a better effect with acceptable safety profile in preventing CINV prompted by the three-day cisplatin-based HEC regimen during the whole cycle.

### Supplementary Information

Below is the link to the electronic supplementary material.

Supplementary file1 (DOCX 21 KB)

## Data Availability

We declare that the manuscript reports an original clinical trial, and the data underlie this study can be obtained from the corresponding author if you have probable reason.
