# Supplementary material for: Two doses of fosaprepitant included prophylactic treatment for the three-day cisplatin-based chemotherapy induced nausea and vomiting
Source: J Cancer Res Clin Oncol. 2024 Jun 5;150(6):290. doi: 10.1007/s00432-024-05766-7 (PMC11153275; doi:10.1007/s00432-024-05766-7)
Supplement: Supplementary file 1 — Supplementary file1 (DOCX 21 KB) [file 432_2024_5766_MOESM1_ESM.docx]

**A randomized controlled trial about the antiemetic effect of prolonged administration of fosaprepitant for lung cancer patients receiving 3 days cisplatin-based chemotherapy**

**Study Description**

Brief Summary:

This is a multicenter, randomized, parallel-group, open-labelled, phase III study assessing

the safety and antiemetic efficacy of two doses of fosaprepitant included prophylactic treatment versus one dose for the three-day cisplatin-based chemotherapy induced nausea and vomiting.

| Condition or disease | Intervention/treatment | Phase |
| --- | --- | --- |
| lung cancer | Drug: Fosaprepitant 150mg iv on day 1 and day 3 plus oral palonosetron plus oral dexamethasone  Drug: Fosaprepitant 150mg iv on day 1 plus oral palonosetron plus oral dexamethasone | Phase 3 |

Detailed Description:

In NCCN, CSCO and other major antiemetic guidelines, it is recommended that the use of strong emetic chemotherapeutic drugs should be given triple antiemetic therapy including substance P neurokinin 1 (NK1) receptor antagonists. Fosaprepitant is a commonly used NK1 receptor antagonist. The guidelines recommend that patients who receive one-day cisplatin chemotherapy should be given fosaprepitant to prevent chemotherapy induces nausea and vomiting (CINV) on the first day of chemotherapy. However, there is no clear guidance on the duration of antiemetic drugs used in multi-day chemotherapy. Multiple days cisplatin containing regimen was widely used in various malignancies. Should fosaprepitant be given longer to do a better prevention of nausea and vomiting during the whole chemotherapy cycle? In order to answer this question, we designed this trial to explore the safety and antiemetic efficacy of two doses of fosaprepitant included prophylactic treatment for the three-day cisplatin-based chemotherapy induced nausea and vomiting.

**Study Design**

Study Type: Interventional (Clinical Trial)

Estimated Enrollment: 204 participants (study group:102; control group:102)

Allocation: Randomized

Intervention Model: Parallel Assignment

Masking: None (Open Label)

Primary Purpose: Supportive Care

Official Title: A randomized controlled trial about the antiemetic effect of prolonged administration of fosaprepitant for lung cancer patients receiving 3 days cisplatin-based chemotherapy

Estimated Study Start Date: February, 2021

| **Group** | **Intervention/treatment** |
| --- | --- |
| Group 2DF: Fosaprepitant 150mg once on days 1 and 3 | Drug: Fosaprepitant 150mg iv on day 1 and day 3 plus oral palonosetron plus oral dexamethasone  fosaprepitant 150mg iv one hour before cisplatin on day 1 and day 3, oral palonosetron 0.5mg on day 1, and oral dexamethasone 6mg on day 1, 3.75mg on day 2-4. |
| Group C: Fosaprepitant 150mg on day 1 | Drug: Fosaprepitant 150mg iv on day 1 plus oral palonosetron plus oral dexamethasone  fosaprepitant 150mg iv one hour before cisplatin on day 1, oral palonosetron 0.5mg on day 1, and oral dexamethasone 6mg on day 1, 3.75mg on day 2-4 |

**Outcome Measures**

Primary Outcome Measures

1. The safety of two doses of fosaprepitant included prophylactic treatment for the three-day cisplatin-based chemotherapy induced nausea and vomiting.

2. None CINV days (NCDs).

Secondary Outcome Measures

1. Total control of CINV in the acute phase, the delayed phase and beyond the risk phase.

2. Time to attain total control of CINV.

3. Patients’ life function status.

4. The time of appetite recovery.

5. No impact on daily life (NIDL) (defined as total score > 108 by the FLIE questionnaire).

**Eligibility Criteria**

Inclusion criteria

1. Patients with lung cancer confirmed by pathology (including NSCLC and SCLC);
2. Aged from 18 to 75 years;
3. ECOG physical condition score was 0-2;
4. Received chemotherapy for the first time, the chemotherapy regimen was cisplatin combined with one of the following drugs: paclitaxel, albumin paclitaxel, gemcitabine, vinorelbine, pemetrexed, etoposide;
5. Cisplatin administration regimen was given for three days;
6. Within 21 days after the beginning of the first cycle of chemotherapy, there was no radiotherapy, immunotherapy or targeted treatment plan (except bevacizumab and Endostar).
7. No gastrointestinal and intestinal metastases, no brain metastases;
8. No history of chronic gastrointestinal diseases leading to recurrent nausea and vomiting, no history of chronic nausea and vomiting (such as vertigo, Meniere syndrome);
9. The indexes of hematology, liver and kidney function and important organ function met the requirements of chemotherapy;
10. The estimated survival time was more than 3 months;
11. Patients and their spouses were willing to take effective contraceptive measures;
12. Patients participated in the study voluntarily and were able to follow planned study visits, treatment plans, laboratory tests, and other research steps.

Exclusion criteria：

1. Currently receiving treatment in other therapeutic clinical studies;
2. Had received systemic chemotherapy in the past;
3. Subjects who have not yet controlled stable cancer pain, need to titrate cancer pain or adjust the dose of opioid painkillers, or the digestive tract reaction of opioids has not been completely controlled;
4. Nausea and vomiting caused by whatever reason occurred within three days;
5. There is a history of active gastrointestinal ulcer and gastrointestinal bleeding within one year;
6. The subjects who could not read or could not complete the questionnaire independently;
7. No oral drugs;
8. Antiemetic drugs were given within 24 hours before chemotherapy, except for chemotherapy pretreatment, such as dexamethasone and diphenhydramine pretreatment before paclitaxel chemotherapy;
9. Patients with poor compliance and are not expected to complete the study as planned;
10. Patients who are known to be allergic to research drugs or ingredients;
11. Situations where other researchers believe that they cannot be included in the group.

**More Information**

Registration Platform: Chinese Clinical Trial Registry

Registration Number: ChiCTR2100042665

Date of Registration: 2021-01-26

Study leader: Yanying Li, West China Hospital, Sichuan University

Name of the Ethic Committee: Ethics Committee on Biomedical Research, West China Hospital of Sichuan University

Study Execute Time: From February 1, 2021 to December 31, 2022

Primary sponsor: West China Hospital, Sichuan University

Primary sponsor's address: 37 Guoxue Lane, Wuhou District, Chengdu, Sichuan

Collaborator:

1. Sichuan Cancer Hospital

2. Sichuan Provincial People's Hospital

3. Chengdu Seventh People's Hospital

4. Chengdu First People's Hospital

5. Chengdu Fifth People's Hospital

6. Panzhihua Central Hospital

7. Affiliated Hospital of North Sichuan Medical College

8. The Affiliated Hospital of Southwest Medical University

9. Dachuan District People's Hospital

10. The 903th Hospital
